# Targeted therapy for immune mediated skin diseases. What should a dermatologist know?^☆^

**DOI:** 10.1016/j.abd.2023.10.002

**Published:** 2024-03-22

**Authors:** Edinson López, Raúl Cabrera, Cristóbal Lecaros

**Affiliations:** Department of Dermatology, Facultad de Medicina Universidad del Desarrollo-Clínica Alemana de Santiago, Santiago, Chile

**Keywords:** Atopic dermatitis, Biologics, Immune modulators, Inflammatory skin diseases, Jak inhibitors, Psoriasis, Targeted therapy

## Abstract

**Background:**

Molecularly targeted therapies, such as monoclonal antibodies (mAbs) and Janus Kinase inhibitors (JAKis), have emerged as essential tools in the treatment of dermatological diseases. These therapies modulate the immune system through specific signaling pathways, providing effective alternatives to traditional systemic immunosuppressive agents. This review aims to provide an updated summary of targeted immune therapies for inflammatory skin diseases, considering their pathophysiology, efficacy, dosage, and safety profiles.

**Methods:**

The review followed the Preferred Reporting Items for Systematic Reviews and Meta-analyses (PRISMA) guidelines. A systematic search was conducted on PubMed over the past 10 years, focusing on randomized clinical trials, case reports, and case series related to targeted immune therapies in dermatology. Eligibility criteria were applied, and data were extracted from each study, including citation data, study design, and results.

**Results:**

We identified 1360 non-duplicate articles with the initial search strategy. Title and abstract review excluded 1150, while a full-text review excluded an additional 50 articles. The review included 143 studies published between 2012 and 2022, highlighting 39 drugs currently under investigation or in use for managing inflammatory skin diseases.

**Study limitations:**

The heterogeneity of summarized information limits this review. Some recommendations originated from data from clinical trials, while others relied on retrospective analyses and small case series. Recommendations will likely be updated as new results emerge.

**Conclusion:**

Targeted therapies have revolutionized the treatment of chronic skin diseases, offering new options for patients unresponsive to standard treatments. Paradoxical reactions are rarely observed. Further studies are needed to fully understand the mechanisms and nature of these therapies. Overall, targeted immune therapies in dermatology represent a promising development, significantly improving the quality of life for patients with chronic inflammatory skin diseases.

## Introduction

Molecularly targeted therapies have become an important tool for treating dermatological diseases.^1^

The main groups of targeted therapy for inflammatory skin diseases are Monoclonal Antibodies (mAbs) and Janus Kinase Inhibitors (JAKis), but there are also fusion proteins produced by recombinant DNA such as etanercept. Formerly referred to as biologics – since they were synthesized as products of living organisms – today these therapies are also made up of small molecules so the term targeted therapy should be preferred.^2^ Also, the term targeted immune modulators is used in reference textbooks.^3^

The mechanism of action is heterogeneous among the different molecules, but all of them modulate the immune system through stimulatory or inhibitory drives, acting at specific points of the signaling pathways of inflammation.^4^ Their effectiveness and safety profile often are homologous to standard systemic immunosuppressive agents or even better. Like all new drugs, they are not free of adverse events and their cost represents an important barrier for their accessibility.^5^

**Fig. 1:**
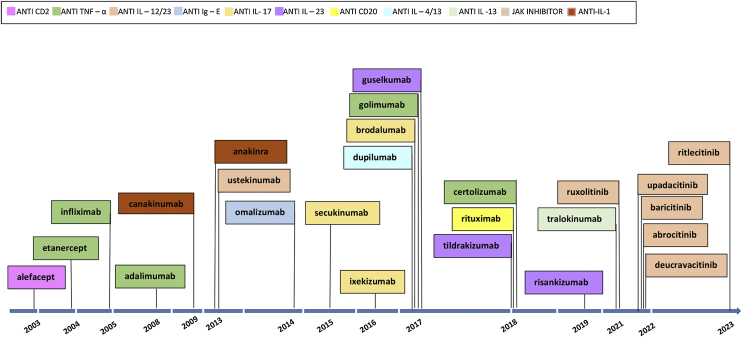
Approved treatments of targeted immune therapies by the FDA for inflammatory skin diseases.

The first examples of targeted immune therapies for inflammatory diseases in dermatology were the clinical trials of alefacept,^6^ efalizumab,^7^ etanercept^8^ and infliximab^9^ for psoriasis in the early 2000s. Alefacept and efalizumab have been replaced by newer molecules with a constant renewal of targeted immune therapies and therapy drugs for different inflammatory skin diseases (Fig. 1).

This narrative review aims to update, summarize, and prioritize the large quantity of information about critical inflammatory skin diseases in a handy clinical format. We suggest indications for use considering current data on the efficacy, dose, and safety profile of targeted immune therapy.

## Material and methods

This review was reported based on the Preferred Reporting Items for Systematic Reviews and Meta-analyzes (PRISMA) guidelines.^10^ We did a systematic search on Pubmed in the last 10 years (January 2012 to July 2023). (Search strategy, Supplementary Material 1). We also screened the references of all included studies to identify any additional eligible studies. Systematic reviews and Randomized Clinical Trials (RCT) in English were selected. We also included case reports and case series if they were conducted on human participants. Studies were excluded if the biological product used was in the context of alternative medicine or the quality of the report was insufficient.

### Data extraction

The titles and abstracts of all records were independently screened for eligibility by two authors (E.L. and C.L.). The full texts of papers deemed potentially eligible were critically appraised and assessed for eligibility. Any disagreement on the inclusion or exclusion of a paper between the two investigators was reviewed by a third investigator (R.C.) to reach a consensus. Data extracted from each study included citation data (title of the study, authors, publication year), study design (study aim, period, setting), and results (demography of the population, outcome measures).

## Results

### Literature search

**Fig. 2:**
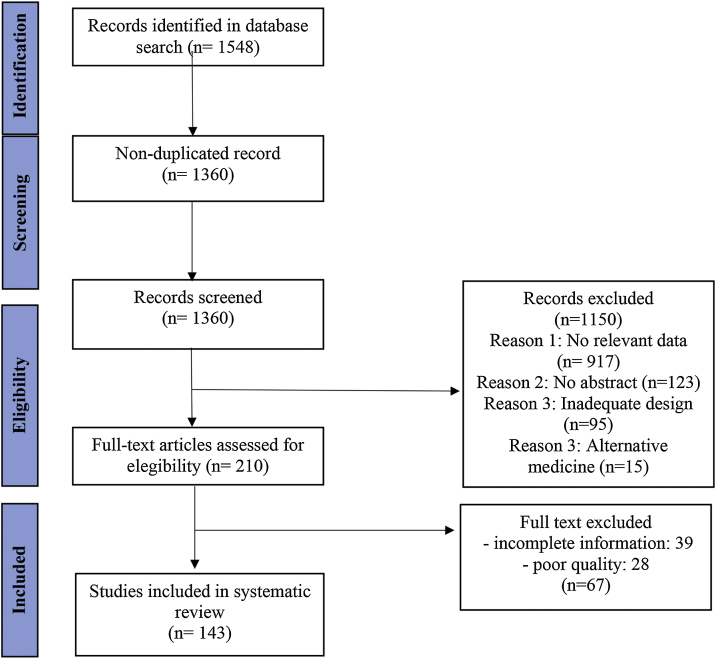
PRISMA Flowchart.

We identified 1360 non-duplicate articles with the initial search strategy. Title and abstract review excluded 1150, while a full-text review excluded an additional 50 articles. One hundred and forty-three studies were included in this review, as outlined in the PRISMA flow diagram (Fig. 2).

### Psoriasis

Psoriasis is a chronic, immune-mediated skin disease that affects approximately 1% of the population worldwide.^11^, ^12^, ^13^ Despite compelling evidence supporting the use of targeted immune therapies, the selection of a specific drug can be challenging.^14^ The final decision for treatment should include the patient’s preference and eventually consultation with physicians of other specialties to balance risks and benefits.

#### Eligibility criteria

Patients should be considered for targeted immune therapy in a variety of circumstances: if severe psoriasis,^15^, ^16^, ^17^ defined as involvement of greater than 10% of the total Body Surface Area (BSA), a PASI or a DLQI scores greater than 10 or compromised of specific locations (hands, feet, scalp, face, or genital area), when psoriasis causes intractable pruritus or has serious emotional consequences with a disease duration greater than 6-months,^15^ or is unresponsive to conventional therapy, or cannot receive standard systemic therapy due to patient’s general condition, comorbidities, or has a severe disease such as erythrodermic, pustular psoriasis or psoriatic arthritis.^15^, ^16^, ^17^

#### Therapy

There are multiple targeted immune therapy for psoriasis. Targeted therapies like anti-TNF-α, anti‐IL17, anti-IL23, and anti‐IL12/23 can reach PASI-90 in more patients than previous systemic treatment (i.e., cyclosporin and methotrexate).^18^ Anti‐IL17 (i.e., ixekizumab, secukinumab, and brodalumab) and anti‐IL23 (risankizumab, tildrakizumab and guselkumab) treatments showed a higher proportion of patients reaching PASI 90 compared to all the systemic interventions evaluated in the last network meta-analysis.^18^

TNF-α inhibitors were the first biologics drugs approved for the treatment of psoriasis.^12^, ^19^ Meta-analysis shows that among this class infliximab has the highest efficacy, followed by certolizumab, adalimumab, and etanercept.^18^, ^19^ The FDA approved sequentially etanercept (2004), infliximab (2005), adalimumab (2008), golimumab (2017), and certolizumab (2018).

IL-17 inhibitors reach a total clearance of disease (i.e. PASI-100) in about half of the patients^11^, ^12^ and have the fastest onset of action. The drugs approved by FDA to treat psoriasis are secukinumab (2015), ixekizumab (2016), and brodalumab (2017).

IL-23 inhibitors risakuzumab and guselkumab have recently demonstrated a better efficacy and safety profile compared to anti-IL-17 and a convenient dosing regimen.^20^, ^21^ The drugs approved by FDA are guselkumab (2017), tildrakizumab (2018) and risankizumab (2019). Ustekinumab (2013) is a mAbs that inhibits IL-12 and IL-23.

JAKis (deucravacitinib, peficitinib, baricitinib, solcitinib, itacitinib, abrocitinib, brepocitinib, ruxolitinib and tofacitinib) have also been used for the treatment of psoriatic patients.^22^, ^23^ Although we still lack complete data on their final clinical impact on psoriasis treatment, recent trials are showing a better response than certain conventional therapies (e.g., methotrexate, cyclosporine) but inferior to targeted immune therapy (e.g., risankizumab).^18^ Deucravacitinib^24^, ^25^ a selective allosteric Tyrosine Kinase 2 (TYK2) inhibitor is the only JAKi approved by the FDA (2022), achieving a 70% PASI-75 at week 24.

In recent years (2004 to 2020), 51 clinical guidelines addressing the management of psoriasis have been published, and 41 of these mention the use of biologics.^26^ During 2021–2022 several dermatological societies have updated their clinical guidelines considering the approval of new molecules by drug regulatory agencies.^27^, ^28^, ^29^, ^30^, ^31^, ^32^, ^33^ Guidelines recognize anti-IL-23 and anti-IL-17 drugs as part of the first-line treatment profile for psoriasis management. A major concern results from their high cost and accessibility. The final decision for the selection of a drug for the treatment of a psoriatic patient depends on several clinical and epidemiological factors (Table 1).

**Table 1:** Targeted immune therapies for psoriasis.

Drug	Mechanism of action	Dosage and via of administration	Indication	Efficacy	FDA	Level of evidence	Grade of recommendation
IL-23							
Risankizumab	Anti-IL-23	Induction dose: 150 mg weeks 0 and 4/SC	Moderate to severe plaque psoriasis (>10% PASI, BSA or DLQI)	74% PASI 90 week 12^25^, ^26^	Yes	I	A
		Maintenance dose: 150 mg every 12 weeks/SC					
Guselkumab	Anti-IL-23	Induction dose: 100 mg weeks 0 and 4/SC	Moderate to severe plaque psoriasis (>10% PASI, BSA or DLQI)	68% PASI 90 week 12^25^, ^26^	Yes	I	A
		Maintenance dose: 100 mg every 8 weeks/SC					
Tildrakizumab	Anti-IL-23	Induction dose: 100 mg weeks 0 and 4/SC	Moderate to severe plaque psoriasis (>10% PASI, BSA or DLQI)	39% PASI 90 week 12^25^, ^26^	Yes	I	A
		Maintenance dose: 100 mg every 12 weeks/SC					
IL-17							
Brodalumab	Anti-IL-17	Induction dose: 210 mg weeks 0, 1 and 2/SC	Moderate to severe plaque psoriasis (>10% PASI, BSA or DLQI)	73% PASI 90 week 12^25^, ^26^	Yes	I	A
		Maintenance dose: 210 mg every 2 weeks/SC					
Ixekizumab	Anti-IL-17	Induction dose: 160 mg week 0 SC, 80 mg weeks 2, 4, 6, 8, 10, 12/SC	Moderate to severe plaque psoriasis (>10% PASI, BSA or DLQI)	72% PASI 90 week 12^25^, ^26^	Yes	I	A
		Maintenance dose: 80 mg every 4 weeks/SC					
Secukinumab	Anti-IL-17	Induction dose: 300 mg weeks 0,1,2,3 and 4/SC	Moderate to severe plaque psoriasis (>10% PASI, BSA or DLQI)	60% PASI 90 week 12^25^, ^26^	Yes	I	A
		Maintenance dose: 300 mg every month/SC					
IL-12/23							
Ustekinumab	Anti-IL-12/23	Induction dose: <100 kg: 45 mg weeks 0 and 4/SC; >100 kg: 90 mg weeks 0 and 4/SC	Moderate to severe plaque psoriasis (>10% PASI, BSA or DLQI)	Adults: 42% PASI 90 week 12^25^, ^26^	Yes	I	A
		Maintenance dose: <100 kg: 45 mg weeks every 12-weeks/SC; >100 kg: 90 mg every 12-weeks/SC					
TNF-a							
Infliximab	Anti-TNF-α	Induction dose: 5 mg/kg weeks 0, 2 and 6/IV	Moderate to severe plaque psoriasis (>10% PASI, BSA or DLQI)	53% PASI 90 week 12^25^, ^26^	Yes	I	A
		Maintenance dose: 5 mg/kg every 8 weeks/IV					
Adalimumab	Anti-TNF-α	Induction dose: 80 mg week 0, 40 mg week 1/SC	Moderate to severe plaque psoriasis (>10% PASI, BSA or DLQI)	41% PASI 90 week 12^25^, ^26^	Yes	I	A
		Maintenance dose: 40 mg every 2 weeks/SC					
Certolizumab	Anti-TNF-α	Induction dose: 400 mg weeks 0, 2 and 4/SC	Moderate to severe plaque psoriasis (>10% PASI, BSA or DLQI)	40% PASI 90 week 12^25^, ^26^	Yes	I	A
		Maintenance dose: <90 kg: 200 mg every 2-weeks/SC; >90 kg: 400 mg every 2-weeks/SC					
Golimumab	Anti-TNF-α	Induction dose:200 mg week 0, 100 mg week 2	Moderate to severe plaque psoriasis (>10% PASI, BSA or DLQI)	40% PASI 75 in week 14^25^	No	I	A
		Maintenance dose: 50 mg mg every 4 weeks/SC					
Etanercept	Anti-TNF-α	Induction dose: 50 mg twice a week/SC for 12 weeks	Moderate to severe plaque psoriasis (>10% PASI, BSA or DLQI)	Adults: 23% PASI 90 week 12^25^, ^26^	Yes	I	A
		Maintenance dose: 50 mg once weekly/SC					
JAK-2i							
Deucravacitinib	JAK-2 inh	6 mg once daily/VO	Moderate to severe plaque psoriasis (>10% PASI, BSA or DLQI)	58% PASI 75 week 16^25^, ^30^, ^31^, ^32^	Yes	I	A

SC, Subcutaneous; VO, Orally; IV, Intravenous; yo, years old; PASI, Psoriasis Area and Severity Index; BSA, Body Surface Area; DLQI, Dermatology Life Quality Index.

#### The safety profile of targeted immune therapy for psoriasis

The safety profile of targeted immune therapy for psoriasis is well described, the most common adverse events being upper respiratory tract infections and injection site reactions for all drug classes.^34^, ^35^ General considerations should be taken concerning reactivation of tuberculosis, hepatitis B and C. The usage of JAKis should also consider previous infections by viruses of the Herpes family.^36^, ^37^

Current evidence shows that targeted immune therapy does not increase the risk of major adverse cardiovascular events such as myocardial infarction, cerebrovascular accident cardiovascular death, or the incidence of malignancies.^35^, ^37^, ^38^, ^39^

A common cutaneous adverse reaction is the phenotypic switch from psoriasis to atopic eczema that occurs in up to 1%–12.1% of patients taking anti-TNF-α or anti-IL-17/IL-23 drugs.^40^ Topical treatment was successful in some cases, but in others, the biologic treatment needs to be discontinued.

A paradoxical reaction – psoriatic worsening and/or new psoriatic lesions – has been reported with the use of certain drugs (e.g., anti-TNF-α drugs and ustekinumab). These reactions usually do not require cessation of therapy and are self-limited.^37^ Other relevant adverse reactions with anti-TNF-α class are urinary tract infection, back pain, arthralgia, pruritus and erysipelas, invasive fungal infections, lymphoma, heart failure, cytopenia, induction or exacerbation of demyelinating disease and lupus-like syndrome (e.g., infliximab).^40^

Anti-IL-17 and anti-IL-12/23 drugs have less data about their safety profile. An adverse reaction of interest in IL-17 and IL-12/23 inhibitors is mucocutaneous candidiasis (1.7% in patients treated with secukinumab, 3.3% ixekizumab, 4.0% brodalumab, 2.3% ustekinumab).^41^ Episodes of exacerbation of inflammatory bowel disease have also been observed using these drugs. Patients with a history of Crohn's disease or ulcerative colitis should be treated with another targeted immune therapy.^37^

For JAKis the most common adverse effects were herpes simplex reactivation, mouth ulcers, pneumonia, COVID-19, malignancies including lymphoma, rhabdomyolysis, increased creatine phosphokinase, increased triglycerides, and increased transaminases.^23^

With the available data the, rates of any adverse effect were the lowest for tildrakizumab, certolizumab, and etanercept.^34^ For serious AE the lowest were for certolizumab, risankizumab, and etanercept. In the long-term treatment, risankizumab had the most favorable benefit-risk profile.^34^

#### Special populations

Pregnancy: the only FDA-approved mAbs for use in pregnant patients is certolizumab pegol. It is a PEGylated Fab mAb directed against TNF-α that does not cross the placental membrane.^12^, ^19^

Pediatric: The mAbs authorized by the FDA for children over 4 years of age are etanercept and adalimumab, secukinumab from 6 years, and ustekinumab from 12 years old.^18^, ^19^, ^42^

### Atopic dermatitis

Atopic dermatitis (AD) is a chronic relapsing inflammatory skin disease that affects 2%–5% of adults and 20% of children.^43^ The efficacy of targeted immune therapy in AD has been proven by multiple studies. A recent systematic review of 60 clinical trials conducted in 16,579 patients confirms the clinical efficacy of these targeted therapies in AD.^44^

#### Eligibility criteria

Both children and adults can be considered for targeted immune therapy if they have severe disease, defined as; involvement of greater than 10% of the total BSA, Investigator Global Assessment (IGA) of at least 3, Eczema Area and Severity Index (EASI) ≥16, Scoring Atopic Dermatitis (SCORAD) ≥50, and Peak Pruritus Numerical Rating Scale (PP-NRS) severity score ≥4, or when it causes intractable pruritus or it has serious emotional consequences.^45^, ^46^

#### Therapy

Multiple targeted therapies are available for AD that must be selected according to different clinical criteria in each patient (Table 2). Dupilumab has demonstrated similar efficacy to higher-dose cyclosporine in patients older than 6 months with a more reliable response and superiority to methotrexate and azathioprine with considerably fewer side effects.^44^ This has made targeted immune therapy very promising for AD, but the comparison of efficacy and safety between targeted therapies is difficult.^44^, ^47^ Four systemic drugs have been approved by the FDA (dupilumab,^48^ tralokinumab,^49^ abrocitinib,^50^ upadacitinib^51^) and other drugs are used off-label or in clinical trials.

**Table 2:** Targeted immune therapies for atopic dermatitis.

Drug	Mechanism of action	Dosage and via of administration	Indication	Efficacy	FDA	Level of evidence	Grade of recommendation
Upadacitinib	JAK 1	15–30 mg once daily/VO	Severe AD (BSA ≥ 10%, IGA ≥ 3, EASI ≥ 16, SCORAD ≥ 50, PP-NRS ≥ 4) refractory to other treatments	71% EASI 75 week 16 (30 mg)^43^	Yes	I	A
Abrocitinib	JAK 1	100–200 mg once daily/VO	Severe AD (BSA ≥ 10%, IGA ≥ 3, EASI ≥ 16, SCORAD ≥ 50, PP-NRS ≥ 4) refractory to other treatments	70% EASI 75 week 16 (200 mg)^50^	Yes	I	A
Dupilumab	Anti-IL-4/13	Induction dose: 600 mg single dose/SC	Severe AD (BSA ≥ 10%, IGA ≥ 3, EASI ≥ 16, SCORAD ≥ 50, PP-NRS ≥ 4) refractory to other treatments	64% EASI 75 week 16^51^	Yes	I	A
		Maintenance dose: 300 mg every 2 weeks/SC					
Lebrikizumab	Anti-IL-13	Induction dose: 500 mg week 0 and 2/SC	Severe AD (BSA ≥ 10%, IGA ≥ 3, EASI ≥ 16, SCORAD ≥ 50, PP-NRS ≥ 4) refractory to other treatments	58,8% EASI 75 at week 16^55^	No	I	A
		Maintenance dose: 250 mg every 2 weeks/SC					
Baricitinib	JAK 1/2	4 mg once daily/VO	Severe AD (BSA ≥ 10%, IGA ≥ 3, EASI ≥ 16, SCORAD ≥ 50, PP-NRS ≥ 4) refractory to other treatments	54% EASI 75 at week 16^60^	No	I	A
Tralokinumab	Anti-IL-13	Induction dose: 600 mg single dose/SC	Severe AD (BSA ≥ 10%, IGA ≥ 3, EASI ≥ 16, SCORAD ≥ 50, PP-NRS ≥ 4) refractory to other treatments	25% EASI 75 week 16^48^	Yes	I	A
		Maintenance dose: 300 mg every 2 weeks/SC					
Etokimab	Anti-IL-33	300 mg Week 0, 150 mg weeks 4, 8 and 12/IV	Severe AD (BSA ≥ 10%, IGA ≥ 3, EASI ≥ 16, SCORAD ≥ 50, PP-NRS ≥ 4) refractory to other treatments	33% EASI 75 at week 4^65^	No	II	B
Fezakinumab	Anti-IL-22	Induction dose: 600 mg single dose/SC	Severe AD (BSA ≥ 10%, IGA ≥ 3, EASI ≥ 16, SCORAD ≥ 50, PP-NRS ≥ 4) refractory to other treatments	SCORAD decline of 14 points at week 12^63^	No	II	B
		Maintenance dose: 300 mg every 2 weeks for 10 weeks/SC					
Nemolizumab	Anti-IL-31	30 mg every 4 weeks/SC	Severe AD (BSA ≥ 10%, IGA ≥ 3, EASI ≥ 16, SCORAD ≥ 50, PP-NRS ≥ 4) refractory to other treatments	69% EASI improvement week 12 (30 mg)^64^	No	II	B
Tezepelumab	Anti-TSLP	280 mg every 2 weeks for 3 months/SC	Severe AD (BSA ≥ 10%, IGA ≥ 3, EASI ≥ 16, SCORAD ≥ 50, PP-NRS ≥ 4) refractory to other treatments	65% EASI 50 week 12^62^	No	II	B
Topicals							
Tofacitinib	JAK 1/3	2% topical, once daily	IGA 2–3, BSA 2−20%	82% EASI improvement at week 4^67^	No	II	B
Ruxolitinib	JAK 1/2	1,5% topical twice daily	IGA 2–3, BSA 2%–20%	71% EASI improvement at week 4^66^	Yes	I	A

SC, Subcutaneous; VO, Orally; IV, Intravenous; EASI, Eczema Area and Severity Index; BSA, Body Surface Area; IGA, Investigator Global Assessment; SCORAD, SCORing Atopic Dermatitis, PP-NRS, Peak Pruritus Numerical Rating Scale; AD, Atopic Dermatitis.

Compared with dupilumab, abrocitinib, 200 mg daily, and upadacitinib, 30 mg daily, were associated with higher reductions in EASI scores meanwhile baricitinib and tralokinumab, reductions in EASI scores were similar or slightly worse than dupilumab.^44^

Dupilumab is an anti-IL-4/13 mAb approved by the FDA in 2017 for the treatment of moderate to severe AD from 6 months.^52^ The therapeutic effect of dupilumab is primarily mediated through its inhibition of the alpha subunit of the Interleukin-4 Receptor (IL-4Rα).^53^, ^54^

Tralokinumab is an anti-IL-13 mAb approved by the FDA in 2021 for the treatment of moderate-to-severe AD in adults. In 2 RCTs, tralokinumab 300 mg every 2 weeks achieved an EASI 75 in 25%–33.2% of patients at 16 weeks of treatment. These RCTs also showed early improvements in pruritus, sleep interference, and DLQI. Patients maintained these responses after 52 weeks of treatment with tralokinumab without any rescue medication.^49^, ^55^ Lebrikizumab, a mAb that targets IL-13 in two randomized, double-blind, placebo-controlled, phase 3 trials in >12-years old patients with moderate to severe AD showed an EASI-75 response in 58.8% and 52.1% of patients respectively at week 16.^56^

Abrocitinib is a JAK-1 inhibitor approved by the FDA for the treatment of AD in adults in 2022. In moderate to severe AD, abrocitinib showed consistent responses to treatment and presented no new safety concerns compared with dupilumab.^46^, ^57^, ^58^, ^59^ It is used at a dose of 200 mg daily, achieving 70% of EASI 75 at week 12 of treatment.^58^

Upadacitinib is a JAK-1 inhibitor approved by the FDA (2022) for the treatment of AD in patients >12 years old. It shows an improvement in EASI 75 of 71% at week 16 with a dose of 30 mg per day orally.^60^

Recently baricitinib, a first-generation inhibitor of JAK 1–2 not yet approved by FDA in AD, has completed phase 2 and 3 RCTs in combination with topical corticosteroids in adults with moderate-to-severe AD. At 16 weeks baricitinib 4 mg provided 54.9% and 59.4% improvement in EASI score in BREEZE-AD1 and BREEZE-AD2 respectively.^61^

Tezepelumab is a human mAb that binds thymic stromal lymphopoietin (TSLP, an epithelial cell-derived cytokine that induces the production of Th2 cytokines, including IL-4, IL-5, and IL-13)^62^ used for the treatment of asthma. It has been tested at doses of 280 mg every 2 weeks in phase 2 studies, achieving an EASI 50 in 65% of patients at week 12 of treatment.^63^ Fezakinumab, a human mAb against IL-22 is in phase 2 studies, induction doses of 600 mg followed by 300 mg every 2 weeks have demonstrated an improvement in IGA score from 12 weeks of use.^64^ Nemolizumab is an experimental anti-IL-31RA mAb in phase 2 studies. It was reported that 42% of patients treated showed an improvement in the EASI score at 12 weeks of use.^65^ Etokimab is another experimental mAb that binds IL-33 in the initial phase of development. At the moment, studies have tested doses of 300 mg IV once, achieving EASI 50 in 100% of the participants at 140 days.^66^

New topical targeted immune therapy for AD has recently appeared as a therapeutic tool. Ruxolitinib 1.5% cream (approved by FDA in 2021) for patients >12 years old shows improvements of 71.6% in EASI score after 4 weeks of use.^67^ Tofacitinib 2% cream had shown improvements of 82% on EASI score after 4 weeks of use and is awaiting FDA approval.^68^, ^69^

#### The safety profile of targeted immune therapy for AD

The most common adverse events were upper respiratory tract infections and injection site reactions for all classes.^44^, ^45^ General considerations should be taken in relation to the reactivation of tuberculosis, and hepatitis B, and in the case of JAKis also consider infections by viruses of the Herpes family.^58^, ^60^ Another common adverse effect in all classes was eye symptoms (conjunctivitis, keratitis, blepharitis, ocular pruritus, dryness and discomfort in the eye). Dupilumab and tralokinumab do not require laboratory monitoring but abrocitinib, baricitinib, and upadacitinib need laboratory follow-up. It can produce creatine phosphokinase elevation, thrombocytopenia, and neutropenia.^45^ Other adverse effects reported for JAKis are nausea, headache, diarrhea, fatigue, acne, abdominal pain, and myalgias.^70^

Recently it has been observed an increased risk of seronegative inflammatory arthritis and psoriasis in patients using dupilumab.^71^ Based on current evidence, it appears that the use of anti-TNF-α for psoriasis and anti-IL-4/13 for atopic dermatitis are inversely related. The dual blockade with dupilumab is associated with psoriasiform diseases, while biologics for psoriasis can cause a phenotypic switch to eczema.^72^

For topicals, mild local irritation was the most common adverse effect.^67^, ^68^

There are insufficient data to determine whether any targeted immune therapy is safer than another for AD. Abrocitinib, 100 mg daily, was associated with 2.6 times the odds of serious adverse events compared with dupilumab, but with a higher dose (i.e., abrocitinib, 200 mg daily) the odds were lower (1.4).^44^

Lower rates of serious adverse events were observed for dupilumab compared with placebo.^44^ Dupilumab is also associated with a non-significant increase in Herpes infection. The reason for a decrease in cutaneous infections, including eczema herpeticum is unknown, but improvement in the skin barrier and microbiome caused by targeted immune therapy may explain this observation.^73^

### Hidradenitis suppurativa

Hidradenitis Suppurativa (HS) is an autoinflammatory disease of the pilosebaceous follicle with a prevalence ranging from 0.05% to 4.1%.^74^ There are at least 9 clinical guidelines that consider the use of biologics in the management of HS.^75^, ^76^, ^77^, ^78^, ^79^, ^80^, ^81^, ^82^, ^83^

#### Eligibility criteria

Management with targeted immune therapy should be considered early in severe cases. International guidelines recommend starting a targeted treatment for recalcitrant moderate-to-severe HS, defined as Hurley stage II‒III, or total abscess and inflammatory-nodule count ≥3 at baseline and an inadequate response to oral antibiotic treatment for at least 90 days.

#### Therapy

There are a few targeted therapies for HS that must be selected according to different clinical criteria in each patient (Table 3). Adalimumab^84^ is the only biologic treatment approved by the FDA for HS (2015) and it is recommended as the first-line biologic drug in all guidelines, making infliximab the second option.^74^ Anakinra and bermekimab may be considered following failure of anti-TNF-α agents but evidence to support their efficacy in HS is limited.^74^

**Table 3:** Targeted immune therapies for hidradenitis suppurativa.

Drug	Mechanism of action	Dosage and via of administration	Indication	Efficacy	FDA	Level of evidence	Grade of recommendation
Adalimumab	Anti-TNF-α	Induction dose: 160 mg day 0, 80 mg day 15/SC	Recalcitrant moderate-to-severe HS (Hurley stage II‒III)	42%‒59% HiSCR improvement in 50% of patients at week 12^83^	Yes	I	A
		Maintenance dose: 40 mg every week/SC or 80 mg every two weeks/SC					
Anakinra	Anti-IL-1	100 mg once daily/SC for 12 weeks	Recalcitrant moderate-to-severe HS (Hurley stage II‒III)	78% of patients showed an HiSCR improvement at week 12^86^	No	II	B
Bermekimab	Anti-IL-1	400 mg every week/SC for 12 weeks	Recalcitrant moderate-to-severe HS (Hurley stage II‒III)	60% of patients showed an HiSCR improvement at week 12^87^	No	II	B
Infliximab	Anti-TNF-α	Induction dose: 5 mg/kg weeks 0, 2 and 6/IV	Recalcitrant moderate-to-severe HS (Hurley stage II‒III)	25%‒50% HSSI improvement at week 8^84^	No	II	B
		Maintenance dose: 5 mg/kg every 8 weeks/IV					

SC, Subcutaneous; IV, Intravenous; HiSCR, Hidradenitis Suppurativa Clinical Response; HSSI, Hidradenitis Suppurativa Severity Index.

Adalimumab showed a 41.8%–58.9% improvement in Hidradenitis Suppurativa Clinical Response (HiSCR) in 50% of patients at 12 weeks.^84^ Infliximab demonstrated a 25%–50% improvement in Hidradenitis Suppurativa Severity Index (HSSI) in 60% of patients at 8 weeks.^85^ Other anti-TNFs are not used to treat HS such as etanercept or golimumab since paradoxical reactions of worsening or inducing the disease.^74^, ^86^

Anti-IL-1 drugs have proven their efficacy in small placebo-controlled-RCT.^87^, ^88^ Anakinra showed a >50% of improvement in Disease Activity Score (DAS) in 78% of patients at 12 weeks of treatment.^87^ Bermekimab showed a 60% of improvement in HiSCR at 12 weeks of treatment.^88^ These drugs should be considered when patients are not candidates for anti-TNF-α therapy.

Clinical guidelines also mention other biologics that have been used in case series successfully for the treatment of HS (guselkumab, risankizumab, secukinumab, bimekizumab, and upadacitinib), but the results in patients who have been treated with these drugs are based mainly on isolated case reports, which is not sufficient to recommend their use in the clinical setting.^86^

#### The safety profile of targeted immune therapy for HS

The safety profile of anti-TNF-α drugs has been discussed in a previous section (see Psoriasis). For anti-IL-1 drugs, the general considerations are similar between them. The most common adverse effects are injection site reactions and upper respiratory tract infections. Other adverse effects reported are headache, abdominal pain, diarrhea, and flu-like symptoms.^89^

### Pyoderma gangrenosum

Pyoderma Gangrenosum (PG) is a neutrophilic dermatosis characterized by chronic and recurrent skin ulcers with a necrolytic border with an estimated incidence of 3–10/1,000,000 cases yearly.^90^ There are currently no FDA-approved drugs for this disease. New targeted immune therapies have been used with some success lately to treat refractory cases of PG, but clinical trials are needed to establish the real efficacy of these therapies.^91^, ^92^, ^93^

#### Eligibility criteria

We suggest considering targeted immune therapy in PG after a thorough study to establish the diagnosis of this condition.^94^ The disease must be recalcitrant or with moderate-severe intensity (BSA > 10%, complex anatomical sites, or excruciating pain).

#### Therapy

There are a few targeted therapies for PG that must be selected according to different clinical criteria for each patient (Table 4). The drug with more data for PG treatment is infliximab.^94^ Infliximab demonstrated improvement of lesions at week 4 (20/29 patients), with complete remission in 21% at week 6.^94^ Infliximab has also been used successfully in combination with other drugs (oral systemic corticosteroids, azathioprine, mycophenolate mofetil and cyclosporine).^92^, ^93^, ^95^

**Table 4:** Targeted immune therapies for pyoderma gangrenosum.

Drug	Mechanism of action	Dosage and via of administration	Indication	Efficacy	FDA	Level of evidence	Grade of recommendation
Infliximab	Anti-TNF-α	Induction dose: 5 mg/kg weeks 0, 2 and 6/IV	Recalcitrant and late stage with moderate-severe disease	68% complete response, 19% partial response^91^	No	I	B
		Maintenance dose: 5 mg/kg every 8 weeks/IV					
Etanercept	Anti-TNF-α	25‒50 mg every week/SC	Recalcitrant and late stage with moderate-severe disease (under research context)	68% complete response, 30% partial response^94^	No	III	C
Adalimumab	Anti-TNF-α	Induction dose: 80 mg week 0/SC	Recalcitrant and late stage with moderate-severe disease (under research context)	77% complete response, 14% partial response^94^	No	III	C
		Maintenance dose: 40 mg every week/SC					
Anakinra	Anti-IL-1	100 mg once daily/SC	Recalcitrant and late stage with moderate-severe disease (under research context)	38% complete response, 21% partial response^90^	No	III	C
Canakinumab	Anti-IL-1β	150‒300 mg every 4−8 weeks/SC	Recalcitrant and late stage with moderate-severe disease (under research context)	55% complete response, 9% partial response^95^	No	III	C
Ustekinumab	Anti-IL-12/23	Induction dose: 90 mg week 0/SC	Recalcitrant and late stage with moderate-severe disease (under research context)	71% complete response, 8% partial response^96^	No	III	C
		Maintenance dose: 45 mg weeks 4 and 8/SC					
Ruxolitinib	JAK 1/2	10 mg twice daily/VO	Recalcitrant and late stage with moderate-severe disease (under research context)	2/2 complete response after 1–15 months^90^	No	III	C

SC, Subcutaneous; VO, Orally.

There are case reports of PG treated with adalimumab and etanercept.^95^ Based on these observational data, no significant differences in the partial or complete response rates to infliximab, adalimumab, and etanercept were found. In a semi-systematic review for TNF-α inhibitors, an 87% partial response rate and a 67% complete response were found.^95^ Corticosteroids and cyclosporine can achieve 90% of partial response rates and only 47% complete response rates after 6 months.^95^

Anti-IL-1 drugs have also been used to treat PG.^91^ There are 29 cases reported in the literature of the use of anakinra in patients with comorbidities such as rheumatoid arthritis, psoriasis, PASH and PAPA syndrome at a dose of 100 mg per day.^91^, ^92^ In an uncontrolled trial canakinumab was used in five steroid-refractory PG patients. Four showed a decrease in target-lesion size and three achieved complete remission.^96^

Other targeted immune therapies used successfully are ustekinumab (anti-IL-12/23)^97^ and ruxolitinib (anti-JAK1-2).^91^, ^92^

#### The safety profile of targeted immune therapy for PG

There are reported cases of paradoxical induction of PG with the use of adalimumab, etanercept and to a lesser extent with infliximab.^90^

Specific adverse reactions for canakinumab are worsening of rheumatoid arthritis, pyrexia, vomiting, sinusitis, and arthralgia and for anakinra weight gain, musculoskeletal pain and vertigo.^96^

### Chronic spontaneous urticaria

Chronic Spontaneous Urticaria (CSU) is defined by the appearance of transient pruritic wheals and/or angioedema for at least 6-weeks without a recognizable trigger.^98^ The global prevalence of CSU is 1.1% according to the Global Burden of Disease.^99^

#### Eligibility criteria

Adults and adolescents 12 years of age and older can be considered for targeted immune therapy if they remain symptomatic despite H1 antihistamine treatment up to 4 times of standard dose, or if they persist symptomatic with combination therapy (H1 antihistamines 4-times the standard dose plus H2-antihistamines and/or leukotriene receptor antagonist).^98^, ^100^

#### Therapy

There is only one targeted immune therapy currently approved for CSU, and other options must be selected in refractory cases or research contexts (Table 5). Omalizumab, an anti-IgE mAb, was approved by the FDA in 2014 for use in patients >12 years old for CSU. This authorization was based on two RCTs (ASTERIA I and ASTERIA II).^101^ RCTs showed that at 40 weeks, 75.3% of CSU patients achieved complete response and 14.6% had partial response.^102^

**Table 5:** Targeted immune therapies for chronic spontaneous urticaria.

Drug	Mechanism of action	Dosage and via of administration	Indication	Efficacy	FDA	Level of evidence	Grade of recommendation
Omalizumab	Anti-IgE	150‒300 mg every 4 weeks /SC	Patients remaining symptomatic despite anti-H1 4× dose	75.3% complete response at 40 weeks^100^	Yes	I	A
		If refractory, consider 600 mg every 4 weeks/SC					
Ligelizumab	Anti-IgE	72‒240 mg every 4 weeks/SC	Patients remaining symptomatic despite anti-H1 4× dose	44% complete response at week 12^102^, ^103^	No	I	B
Secukinumab	Anti-IL-17	Induction dose: 150 mg weeks 0, 1, 2 and 3/SC Maintenance: 150 mg every two weeks/SC	Refractory to omalizumab (under reseach context)	Reduction in UAS7 of 82% at week 12^108^	No	III	C
Dupilumab	Anti-IL-4/13	Induction dose: 600 mg week 0/SC	Refractory to omalizumab (under reseach context)	Improvement in a case series of 6 patients^105^	No	III	C
		Maintenance dose: 300 mg every 2 weeks/SC					
Lirentelimab	Anti-SIGLEC-8	Initial dose: 0.3 mg/kg on day 0	Patients remaining symptomatic despite anti-H1 4× dose (under reseach context)	92% of complete response at 22 weeks^104^	No	I	B
		Subsequent doses: 1 mg/kg on days 29, 57, 85, 113 and 141/IV					
Benralizumab	Anti-IL-5	Induction dose: 30 mg every 4 weeks for 3 doses/SC	Ongoing phase 2 studies in CSU refractory to antiH1	56% of complete response at 20 weeks^106^	No	II	B
		Maintenance dose: 30 mg every 8 weeks/SC					
Mepolizumab	Anti-IL-5	100‒300 mg every 4 weeks/SC	Ongoing phase 2 studies in CSU refractory to antiH1	Ongoing phase 2 studies^106^	No	III	C

SC, Subcutaneous; IV, Intravenous; UAS7, Urticaria Activity Score summed over 7-days.

Ligelizumab an anti-IgE mAb is still under study but recent clinical trials for CSU treatment are showing an efficacy similar to omalizumab.^103^, ^104^

Lirentelimab is an anti-Sialic acid-binding Immunoglobulin-Like Lectin 8 mAb (anti-SIGLEC-8, an inhibitory receptor presents on eosinophils and mast cells). Ligation to Siglec-8 induces eosinophils death, inhibits mast cells IgE-mediated degranulation and *de novo* synthesis of prostaglandin D2. Results of a phase 2 trial with lirentelimab for the treatment of CSU and for the treatment of inducible urticaria demonstrated a 92% complete response at week 22 and only 36% complete response in patients previously refractory to omalizumab treatment.^105^

Dupilumab has demonstrated partial response in case reports.^106^ Benralizumab and mepolizumab, both experimental anti-IL-5R and anti-IL-5 respectively molecules, have also been used.^107^ In both cases treated patients had clinical improvement.^108^ Secukinumab has been used in case reports for the treatment of CSU in refractory patients to H1-antihistaminics and omalizumab treatment, showing a significant reduction in disease activity from baseline.^109^

#### The safety of targeted immune therapy for CSU

The most common but manageable side effects of omalizumab are headache, upper abdominal pain, pyrexia, and local reaction at the injection site. More serious side effects such as anaphylactic reaction, syncope and angioedema have been reported with a frequency of less than 1/1000.^98^

For lirentelimab the most common adverse events included infusion-related reactions, nasopharyngitis, and headache. No treatment-related serious adverse events occurred.^105^

### Alopecia areata

Alopecia Areata (AA) is an autoimmune disease due to the loss of the immune privilege of the hair follicle. Recently FDA approved baricitinib for AA treatment (2022) and many others JAKis are under advanced study for approval.

#### Eligibility criteria

Patients should be considered for JAKis treatment if they have moderate or severe disease (≥30% total scalp hair loss for at least 3–6 months as measured using Severity of Alopecia Tool (SALT) score) or refractory response to conventional treatment for the last 6 months. There is no limitation for previous duration of alopecia and trials have considered patients with a wide range of duration of AA as candidates for JAKis treatment.^110^, ^111^

#### Therapy

There is only one targeted immune therapy currently approved for AA, but other options have been used with success and must be selected according to different clinical criteria in each patient (Table 6). The FDA has approved baricitinib (2022) a JAK 1-2i for the treatment of AA in adults. Its efficacy was evaluated in two randomized, double-blind, placebo-controlled trials (BRAVE AA-1 and BRAVE AA-2).^110^ Patients in these studies received 2 or 4 mg of baricitinib per day. Final results at week 36 showed a 22% adequate response with 2 mg and 38% with 4 mg. Similar results were observed in BRAVE AA-2 (19% adequate response with 2 mg and 35% with 4 mg).^110^ Tofacitinib, a JAK 1-3i has been used to treat moderate to severe cases of AA with >50% of scalp hair loss in adults, achieving complete response in 20% of treated patients and 77% of partial response at 4–18-months of treatment.^112^ In 2023 the FDA approved Ritlecitinib for the treatment of AA in people over 12 years of age based on the results of ALLEGRO, where 23% of patients achieved hair recovery of at least 80% after 6 months of treatment.^113^ Ruxolitinib is the last JAKi used for the treatment if AA achieving overall responses in 93% of patients and complete response in 21% of patients treated at 6 months of follow-up.^111^

**Table 6:** Targeted immune therapies for alopecia areata.

Drug	Mechanism of action	Dosage and via of administration	Indication	Efficacy	FDA	Level of evidence	Grade of recomendation
Baricitinib	JAK 1/2	4 mg once daily/VO	Moderate to severe AA 30% scalp hair loss	35%‒38% adequate hair restorage at week 36^109^	Yes	I	A
Tofacitinib	JAK 1/3	5 mg twice daily/VO	Moderate to severe AA 30% scalp hair loss	20% complete response and 58% adequate hair restorage during 4–18 months of treatment^111^	No	II	B
Ritlecitinib	JAK 3	50 mg once daily/VO	Moderate to severe AA 30% scalp hair loss	23% of patients have >80% of hair restorage at 6-months^112^	Yes	I	A
Ruxolitinib	JAK 1/2	20 mg twice daily/VO	Moderate to severe AA 30% scalp hair loss	21% complete response and 64% adequate hair restorage during 6-months of treatment^110^	No	II	B

VO, Orally.

#### The safety profile of targeted immune therapy for AA

The safety profile of JAK inhibitor drugs has extensively been studied and in general, all JAKis used for AA treatment are well tolerated. Upper respiratory tract infections, hypercholesterolemia, elevated creatine phosphokinase, headache, diarrhea, and nasopharyngitis are the most frequently reported adverse reactions. Less common side effects included other infections such as herpes zoster, herpes simplex, urinary tract infections, and gastroenteritis.^110^, ^111^, ^112^

### Bullous autoimmune diseases

Autoimmune bullous diseases are a group of diseases characterized by production of autoantibodies against adhesion molecules of the skin.^114^ In this review we will discuss targeted immune therapy for Pemphigus Vulgaris (PV), Pemphigus Foliaceus (PF) and Bullous Pemphigoid (BP).

#### Eligibility criteria

Patients are candidates for targeted immune therapy as a first-line option in cases of new-onset moderate to severe PV/PF or previously treated patients who do not achieve clinical remission with systemic corticosteroids or immunosuppressive adjuvants.^114^ The severity of the disease must be measured with the Pemphigus Disease and Area Index (PDAI) score. Multiple mucosal involvement (oral, nasopharyngeal, conjunctival, genital) or a PDAI ≥15 are considered moderate disease. Patients with PDAI score ≥45 or oral lesions, dysphagia and weight loss belong to a severe state of the disease.^115^, ^116^

#### Therapy

Rituximab is the only biologic approved by the FDA (2018) for bullous diseases and is considered the first therapeutic option for PV and PF by international guidelines.^116^ This approval was based on the results of Rituxi3,^115^ in which 89% of pemphigus patients treated with rituximab and oral prednisolone were disease-free and without requirement of any further treatment after 24 months, compared to 34% with prednisolone alone. Rituximab treatment also reduced the dose of corticosteroids required for clinical remission, reducing the side effects associated with high-dose steroids^117^ (Table 7).

**Table 7:** Targeted immune therapies for bullous diseases.

Drug	Mechanism of action	Dosage and via of administration	Indication	Efficacy	FDA	Level of evidence	Grade of recomendation
PV and PF							
Rituximab	Anti-CD20	1g on two occasions 2 weeks apart /IV	Steroid refractory PV and PF	Combined with prednisone 89% complete response at 24 months^114^	Yes	I	A
Efgartigimod	Anti-neonatal receptor for Fc (FcRn)	10‒25 mg weekly for 4 weeks/IV	Steroid refractory PV and PF	Combined with prednisone complete response in 64% patients at 2–41 weeks^118^	No	II	B
Ianalumab	Anti-BLyS	3 mg/Kg IV	Steroid refractory PV and PF (under research context)	73% decrease in mean PDAI score at week 12^120^	No	I	B
BP							
Rituximab	Anti-CD20	375 mg/m^2^ every 1–4 weeks to 500 mg weekly for 2 weeks/IV	Steroid refractory BP	70.5% complete response at 6 months^121^	No	III	C
Omalizumab	Anti-IgE	100‒525 mg every 1–4 weeks/SC	Steroid refractory BP	68% complete response at 6-months^121^	No	III	C
Dupilumab	Anti-IL-4/13	Induction dose: 600 mg/SC single dose	Steroid refractory BP	66.7% complete response at 4.5-months^121^	No	III	C
		Maintenance dose: 300 mg every 2 weeks/SC					

SC, Subcutaneous; IV, Intravenous; PDAI, Pemphigus Disease and Area Index.

Efgartigimod is a human IgG1 antibody targeting the neonatal Fc receptor. The blockade of the neonatal Fc receptor diminishes the availability of pathogenic IgG antibodies and it has been tested for the treatment of PF and PV.^118^ In a phase 2 trial, single use of efgartigimod demonstrated early disease control in 90% of patients after 17 days. Combined with prednisone led to complete clinical remission in 64% of patients after 6 months.^119^ Two phase 3 clinical trials evaluating the efficacy and safety of efgartigimod in adults with pemphigus (NCT04598451 and NCT04598477) are underway.^117^

B-cell Activating Factor (BAFF), also known as B-lymphocyte Stimulator (BLyS) is an important B-cell activator.^120^ Ianalumab is an experimental mAb that binds to BLyS, A phase 2 clinical trial was conducted to determine the doses, benefits, and safety of ianalumab in patients with PV, showing a 73% decrease in mean PDAI score at week 12 in seven patients with pemphigus compared with placebo-treated controls.^121^

Treatment with rituximab, omalizumab and dupilumab is reported in patients with bullous pemphigoid. Cao et al.^122^ conducted a systematic review to evaluate the use of these 3 biologics in patients with BP who had been refractory to the use of other systemic therapies such as systemic corticosteroids, methotrexate, mycophenolate mofetil, azathioprine, cyclosporine and cyclophosphamide. They found that rituximab led to complete remission in 70.5% and partial remission in 23.8% of patients within 6 months of follow-up with a recurrence rate of 20.5%.^122^ Omalizumab led to complete remission in 67.9% and partial remission in 20.8% of patients within 6 months, with a recurrence rate of 5.7%.^122^ Dupilumab led to complete remission in 66.7% and partial remission in 19.4% of patients within 4.5 months of treatment, with a recurrence rate of 5.6%.^123^, ^124^

#### The safety profile of targeted immune therapy for bullous diseases

Although rituximab is an excellent drug for severe PV or PF, it is also associated with well-known severe adverse events. The most well-documented are infusion reactions, infections, cardiac disorders, and cases of fatal progressive multifocal leukoencephalopathy.^114^ Most of these adverse effects can clinically be controlled if they are recognized early stage.

A recent study showed that treatment with rituximab was associated with a lower risk of developing cardiovascular and metabolic comorbidities, so it might be particularly preferred in individuals with cardiovascular and metabolic risk factors, for whom corticosteroid-related adverse events must be strictly avoided.^125^

### Other dermatological diseases

We have considered in this section diseases with scant evidence (small trials or case series, indirect evidence from other diseases or ongoing trials without results) or with topical but no systemic therapy available (Table 8).

**Table 8:** Targeted immune therapies for other diseases.

Diagnostic	Drug	Mechanism of action	Dosage and via of administration	Indication	Efficacy	FDA	Level of evidence	Grade of recomendation
SSJ/TEN	Etanercept	Anti-TNF-α	<65 kg: 25 mg day 0 and 3/SC	Consider use under research context	7.6% mortality^126^	No	III	C
			>65 kg: 50 mg day 0 and 3/SC					
	Infliximab	Anti-TNF-α	5 mg/kg single dose/IV	Consider use under research context	22% mortality ^127^	No	III	C
Lichen planus	Adalimumab	Anti-TNF-α	Induction dose: 80 mg week 0/SC, 40 mg week 1/SC	2^nd^ line in mucosal LP, 3^rd^ line in cutaneous LP	Improvements in itching and healing of skin lesions over a period of 6 months ^133^	No	III	C
			Maintenance dose: 40 mg every 2 weeks SC					
	Ruxolitinib	JAK 1/2	Topical 1.5% twice daily	Cutaneous Lichen Planus	Decrease 7.6 points of mCAILS at 4 weeks ^134^	No	II	B
	Tofacitinib	JAK 1/3	5 mg twice daily/VO	Lichen planopilaris and hypertrophic LP	46% improvement LPPAI at 12 weeks in lichen planopilaris^145^	No	III	C
Cutaneous Lupus Erythematosus	Anifrolumab	Anti-IFN- γ	300 mg/IV every 4 weeks	Cutaneous symptoms in the context of Systemic Lupus Erythematosus	49% of patients achieved reduction in CLASI score of ≥50%^140^	Yes	I	A
Vitiligo	Ruxolitinib	JAK 1/2	1.5% topical twice daily	<10% BSA	75% VASI improvement in 30% of patients at week 24^141^	Yes	I	A
	Tofacitinib	JAK 1/3 inhibitor	2% topical twice daily	<10% BSA	70% VASI improvement at 4 months (sun-exposed areas)^142^	No	III	C
	Tofacitinib	JAK 1/3	5 mg twice daily/VO	>10% BSA	5.4% decrese in BSA in 50% patients^143^	No	II	B
Granuloma annulare	Tofacitinib	JAK 1/3	5 mg bid/VO	Steroid refractory	CR (only case reports)^146^	No	III	C
Sarcoidosis	Tofacitinib	JAK 1/3	5 mg twice daily/VO	Steroid refractory	Improvement in case series^146^	No	III	C
DiHS/DRESS	Tofacitinib	JAK 1/3	5 mg twice daily/VO	Steroid refractory	CR (only case reports) 131, 132	No	III	C
	Etanercept	Anti-TNF-α	50 mg single dose/SC	First line	CR (only case reports) 131, 132	No	III	C
Hypereosinophilic syndrome	Tofacitinib	JAK 1/3	5 mg twice daily/VO	Steroid refractory	CR (only case reports)^147^	No	III	C
	Ruxolitinib	JAK 1/2	25 mg AM-10 mg PM/VO	Steroid refractory	CR (only case reports)^137^	No	III	C
Morphea	Tofacitinib	JAK 1/3	10 mg twice daily/VO	Steroid refractory	Improvement in case report^149^	No	III	C

SC, Subcutaneous; VO, Orally; CR, Complete Response.

#### Stevens-Johnson Syndrome/Toxic Epidermal Necrolysis

Stevens-Johnson Syndrome/Toxic Epidermal Necrolysis (SJS/TEN) is a rare serious mucocutaneous disease caused by exposure to certain drugs, the most frequent being sulfonamides, anticonvulsants, non-steroidal anti-inflammatory drugs, allopurinol, and penicillins.^126^ Due to its low incidence the reported use of biologics in this disease has been limited mostly to case series and only two trials.^126^, ^127^, ^128^ With current data the efficacy and safety of biologic monotherapy and combination therapy cannot be conclusively determined, and the final role of these drugs is still under research.

The largest number of cases reported are related with etanercept. The most used therapeutic regime is 25 mg (in children weighing <65 kg) and 50 mg (adults or children >65 kg) on days 0 and 3. Etanercept monotherapy achieved re-epithelialization in 13 days and a mortality rate of 7.6%, while patients treated with etanercept in combination with corticosteroids achieved re-epithelialization in 11.1 days and had a mortality rate of 7.7%.^126^ Wang et al.^127^ conducted a RCT in which 96 patients with SJS/TEN were enrolled to compare the effects of etanercept to traditional corticosteroids. The study demonstrated that etanercept decreased the TEN-specific severity of illness score (SCORTEN)-based predicted mortality in about 9.4% compared to traditional corticosteroids.^127^ Infliximab was generally used in a dose of 5 mg/kg once.^126^ In case reports of patients treated with infliximab monotherapy the average number of days to reepithelization was 10.4 and there were no cases of mortality reported.^126^ In case reports of TEN treated with the combination infliximab/systemic corticosteroids the average number of days to reepithelization was 14.2 and the average mortality rate was 22.2%. A recent Cochrane review showed that etanercept 25 mg (50 mg if >65 kg) twice weekly until skin lesions healed may reduce disease‐specific mortality compared to corticosteroids (RR = 0.51, 95% CI 0.16 to 1.63) however, the CIs were consistent with possible benefit and possible harm.^129^

Regarding the use of targeted immune therapy used in SJS/TEN compared to corticosteroids, there was no statistical difference observed between the 2 treatment groups in the incidence of adverse events, however, there was a lower incidence of GI hemorrhage reported with biologics versus traditional corticosteroid.^126^, ^127^, ^130^

#### DiHS/DRESS

Drug-induced Hypersensitivity Syndrome/Drug Reaction with eosinophilia and Systemic Symptoms (DiHS/DRESS) is a severe drug reaction with a 10% mortality.^131^ Successful cases have been published using targeted immune therapy. In a recent report studying two cases of DRESS associated with myocardial involvement, tofacitinib 5 mg/bid was used (during 3-years) sequentially associated with corticosteroids, cyclosporin, methotrexate or IVIg.^132^ The two cases demonstrated remission when tofacitinib was utilized.^132^

A previous case report study of 10 patients with DRESS demonstrated a median healing time of 8.5 days after a 50-mg subcutaneous injection of etanercept.^131^ Finally there is a single case report of a patient treated successfully with mepolizumab.^133^

#### Lichen planus

Adalimumab is the only targeted immune therapy recommended by international guidelines for the treatment of cutaneous and mucosal LP, in severe refractory cases.^134^ Its use improves pruritus and accomplishes healing of skin lesions in a period of 6 months.^134^ Another targeted immune therapy for LP is topical ruxolitinib that is in phase 2 studies to treat cutaneous LP.^135^ It has been tested at 1.5% cream twice daily showing clinical and modified Composite Assessment of Index Lesion Severity (mCAILS) score improvement after 4 weeks of treatment.^135^ The use of tofacitinib has been reported in case series at doses of 5 mg (bid) in follow-up periods ranging from 2 to 9 months to treat lichen planopilaris and hypertrophic LP showing clinical and LPAAI score improvement.^136^ The use of other JAKis such as upadacitinib or baricitinib is scarce. There are 13 patients reported in case series and case reports using baricitinib for lichen planopilaris, with 5 achieving partial resolution, 7 having no resolution, and only 1 achieving complete resolution. There are only 2 case reports with the use of upadacitinib for LP both of which achieved complete resolution.^137^ Finally, phase 2 studies are currently being carried out with ixekizumab (cutaneous LP, Lichen planopilaris) and secukinumab (cutaneous LP, Lichen planopilaris and oral LP).^138^

#### Cutaneous lupus erythematosus

The FDA approved anifrolumab for Systemic Cutaneous Lupus (SLE) in July 2021.^139^ Anifrolumab is a fully humanized monoclonal antibody that selectively inhibits the IFN-α receptor 1.^139^ In TULIP-2 RCT anifrolumab was tested for the treatment of SLE, among patients with at least moderately severe skin disease, 49% of patients achieved a reduction in CLASI score of ≥50% compared to 25% in the placebo group.^140^ Belimumab is another drug approved by the FDA in 2011 for the treatment of SLE. We still lack final skin-specific outcomes of this drug in CLE. ^141^

#### Vitiligo

For vitiligo topical ruxolitinib was approved by FDA in 2022 for the treatment of nonsegmental disease compromising <10% of the body’s surface area in adult and pediatric patients >12 years old. In two phase 3 trials (TRuE-V1 and TRuE-V2), subjects were randomized to treatment with ruxolitinib 1.5% cream or placebo twice daily for 2 weeks. At the end of the 24-week treatment period, 30% of ruxolitinib patients had at least 75% improvement in the facial Vitiligo Area Scoring Index (VASI), compared with 10% of placebo patients.^142^ Also in these studies ruxolitinib cream has shown improvement in acral pigmentation, which has historically been a refractory area for repigmentation for phototherapy, topical corticosteroids and tacrolimus.^142^ A pilot study of eleven patients with facial vitiligo using tofacitinib 2% cream in conjunction with narrow band UVB showed a reduction of 70% in VASI score after 2–4 months.^143^ In the other hand a case series of ten patients using tofacitinib 5 mg bid for 10 months and narrow band UVB or photo-exposure had a decrease of only 5.4% in BSA (5 patients) and others (5 patients) did not achieve any repigmentation.^144^

#### Cutaneous granulomatous disorders (granuloma annulare and sarcoidosis)

Granulomatous diseases can be therapeutic challenges. Management may be unsatisfactory with conventional systemic therapy.^145^ Treatment with anti-TNF has been tried with variable results.^146^ JAKis targeted immune therapy appears to be a promising option based on the results of a recent case series.^147^ Tofacitinib 5 mg/bid has been used in patients who were non-responders to corticosteroids. In a case series of only 1 patient complete remission was obtained after 4 months of treatment. For sarcoidosis (3 patients) the same case series showed complete response in 2 patients and 96% improvement in 1 patient.^147^ Further, RCT studies are needed to establish the real value of JAKis in granulomatous diseases.

#### Hypereosinophilic syndrome

A case series of 5 patients refractory to steroids treated with tofacitinib 5 mg/bid showed complete remission in 3 of them and near complete remission in another one.^148^ The remaining patient was unable to complete treatment with tofacitinib due to insurance reasons and treatment was changed to ruxolitinib 25 mg in the morning and 10 mg in the evening. This patient also achieved complete remission.^148^

#### Morphea

There are cases of success using targeted immune therapy for refractory cases of morphea. In two patients with generalized deep morphea associated with eosinophilic fasciitis who did not respond to corticosteroid therapy, tofacitinib (10 mg/bid) was added to phototherapy and methotrexate, achieving clinical improvement in both of them.^149^ Tocilizumab (an anti-IL-6 drug) has been reported to be effective in the treatment of pansclerotic morphea.^150^, ^151^ One 14-year-old girl was treated successfully with infliximab.^150^

## Conclusion

Many targeted immune therapies are currently employed in the management of inflammatory dermatological diseases. Some of these molecules have gained a major role in the treatment of chronic and disabling skin diseases that were not previously controlled with standard treatments, such as anti-IL-4/13 in AD, anti-TNF-α in HS or JAK inhibitors in AA, generating a paradigm shift in the therapeutic armamentarium of dermatology.^44^, ^152^, ^153^ In addition, advances in the understanding of pathophysiology of these diseases have facilitated targeted therapy approaches in inflammatory pathways such as the blockade of JAK-STAT dependent cytokines IL-5, IL-6, IL-10, and IL-13 in DIHS/DRESS.^132^

There are some critical aspects in the development of targeted therapies for inflammatory skin diseases that must be considered. Long-term safety is a relevant aspect of all new drugs. Some adverse effects may remain latent during clinical trials, only appearing with prolonged use. Others – in very few patients – can appear from the beginning of the treatment inducing a paradoxical reaction with a worsening of the clinical condition under treatment-targeted immune therapy (e.g., PG, psoriasis).^34^, ^35^, ^90^ Collaboration between regulatory agencies, healthcare professionals and the pharmaceutical industry is essential in post-marketing surveillance and long-term follow-up studies to evaluate the safety and effectiveness of these new drugs.^154^

For a drug to be considered viable, it must warrant superior clinical outcomes compared to existing treatments. This has led trials to use increasingly better clinometric variables (e.g., PASI-75 vs. PASI-90 in psoriasis),^12^ but not necessarily better comparisons. To compare efficacy between drugs researchers have performed network meta-analyses, which gives some insight but has its limitations.^44^ This missing head-to-head comparison with the old drugs occurs for example in the case of JAKi versus methotrexate in moderate-to-severe atopic dermatitis; JAKi vs. NBUVB in vitiligo, and etanercept versus prednisone in DRESS.^18^, ^44^, ^133^, ^142^ Studies evaluating the economic impact of these decisions are lacking and may help to increase access to these new therapies.^155^

Despite demonstrated efficacy in trials and real-world evidence studies, targeted therapies are still underutilized, especially in regions with lower or middle incomes due to limited availability and inadequate insurance coverage. In Chile, for instance, 92% of the population lacks coverage for targeted therapy for skin conditions, leading to out-of-pocket expenses.^156^ A similar situation is observed in Peru and slightly better conditions in Brazil.^156^ In a survey conducted by the International Psoriasis Council in low-resource or developing countries, including Argentina, Brazil, Chile, Colombia, Mexico, Peru, China, Egypt, Iran, India, and South Africa, dermatologists commonly cited cost as the primary barrier to accessing biological therapy for psoriasis.^157^

Most Clinical Practice Guidelines (CPGs) for skin inflammatory diseases incorporate a logical stratification of the medical management at different stages of the disease.^28^, ^45^, ^75^, ^100^^,^^116^ This stratification may not accurately reflect the socio-health conditions in various regions of the world. The majority (72.1%) of dermatological CPGs originate from countries with a high Sociodemographic Index (SDI), while only a small proportion come from high-middle (8.0%), middle (5.3%), and low-SDI countries (1.8%).^158^ Geographically, the distribution is also nonuniform, corresponding to Europe (51.8%), North America (21.2%), Asia (15.5%), Latin America (4.9%), and Australasia (4.4%).^158^ Thus, CPGs adapted to regional needs in developing countries are still scarce and needed for better management of patients using targeted therapy for skin inflammatory diseases.

These new immune-targeted modulators for the treatment of inflammatory skin diseases are going through the same historical steps of development as many important drugs in use today had previously undergone. The actual data and the high number of new manuscripts appearing daily in the medical literature predict a promising future for the usage of these medications in the treatment of chronic skin conditions and the improvement of the quality of life of our patients.

## Financial support

None declared.

## Authors’ contributions

Edinson Lopez: The study concept and design; data collection, analysis, and interpretation; writing of the manuscript or critical review of important intellectual content; critical review of the literature; final approval of the final version of the manuscript.

Raul Cabrera: The study concept and design; data collection, analysis, and interpretation; writing of the manuscript or critical review of important intellectual content; critical review of the literature; final approval of the final version of the manuscript.

Cristóbal Lecaros: The study concept and design; data collection, analysis, and interpretation; writing of the manuscript or critical review of important intellectual content; critical review of the literature; final approval of the final version of the manuscript.

## Conflicts of interest

None declared.
