# Supplementary material for: Targeted therapy for immune mediated skin diseases. What should a dermatologist know?
Source: An Bras Dermatol. 2024 Mar 22;99(4):546–67. doi: 10.1016/j.abd.2023.10.002 (PMC11221168; doi:10.1016/j.abd.2023.10.002)
Supplement: Supplementary file 1 [file mmc1.docx]

ABD-D-23-00488_Supplementary Material

**Supplementary Material 1** Search strategy.

| Clinical Trial, Randomized Controlled Trial, Systematic Review, in the last 5-years |
| --- |
|  |
| ("Psoriasis"[Mesh]) AND "Biological Products"[Mesh] |
| (psoriasis[Title]) AND (jak inhibitors[Title]) |
|  |
| ("Dermatitis, Atopic"[Mesh]) AND "Biological Products"[Mesh] |
| ("Dermatitis, Atopic"[Title]) AND "jak inhibitors"[Title] |
|  |
| ("Chronic Urticaria"[Mesh]) AND "Biological Products"[Mesh] |
| ("Chronic Urticaria"[Title]) AND "jak inhibitors"[Title] |
|  |
| ("Pyoderma Gangrenosum"[Mesh]) AND "Biological Products"[Mesh] |
| ("Pyoderma Gangrenosum"[Title]) AND "jak inhibitors"[Title] |
|  |
| ("Hidradenitis Suppurativa"[Mesh]) AND "Biological Products"[Mesh] |
| (hidradenitis suppurativa[Title]) AND (jak inhibitors[Title]) |
|  |
| ("Stevens-Johnson Syndrome"[Mesh]) AND "Biological Products"[Mesh] |
| ("Stevens-Johnson Syndrome"[Mesh]) AND (jak inhibitors[Title]) |
|  |
| ("Alopecia Areata"[Mesh]) AND "Biological Products"[Mesh] |
| (alopecia areata[Title]) AND (jak inhibitors[Title]) |
|  |
| ("Pemphigus"[Mesh]) AND "Biological Products"[Mesh] |
| (pemphigus[Title]) AND (jak inhibitors[Title]) |
|  |
| ("Lichen Planus"[Mesh]) AND "Biological Products"[Mesh] |
| (lichen planus[Title]) AND (jak inhibitors[Title]) |
|  |
| ("Cutaneous Lupus Erythematosus"[Mesh]) AND "Biological Products"[Mesh] |
| (Cutaneous Lupus Erythematosus [Title]) AND (jak inhibitors[Title]) |
|  |
| ("Vitiligo"[Mesh]) AND "Biological Products"[Mesh] |
| (vitiligo[Title]) AND (jak inhibitors[Title]) |
|  |
| ("Drug-induced hypersensitivity syndrome/drug reaction with eosinophilia and systemic symptoms "[Mesh]) AND "Biological Products"[Mesh] |
| (Drug-induced hypersensitivity syndrome/drug reaction with eosinophilia and systemic symptoms [Title]) AND (jak inhibitors[Title]) |
|  |
| ("Hypereosinophilic syndrome"[Mesh]) AND "Biological Products"[Mesh] |
| (hypereosinophilic syndrome [Title]) AND (jak inhibitors[Title]) |
|  |
| ("Morphea"[Mesh]) AND "Biological Products"[Mesh] |
| (morphea[Title]) AND (jak inhibitors[Title]) |
